# Micro infarcts are associated with cognitive impairment in neurofibrillary tangle predominant decedents: evidence from the NACC autopsy cohort

**DOI:** 10.1186/s13195-025-01863-y

**Published:** 2025-10-01

**Authors:** Nicko Martinez, Krishna L. Bharani, Saadia Hasan, Cellas A. Hayes

**Affiliations:** 1 Department of Computer Science, Bourns College of Engineering, University of California Riverside, 351 Winston Chung Hall, Riverside, CA 92521 USA; 2 Department of Pathology, Stanford University School of Medicine, 300 Pasteur Drive, Stanford, CA 94305 USA; 3 Department of Epidemiology and Population Health, Stanford University School of Medicine, 1701 Page Mill Road, Palo Alto, CA 94304 USA

**Keywords:** Neurofibrillary tau, Microinfarcts, Alzheimer’s disease continuum, Vascular neuropathology, Cognitive impairment, Neuropathology, Vascular neuropathology

## Abstract

**Background:**

A subset of older adults develops high neurofibrillary tangle burden with minimal amyloid, a biomarker profile consistent with suspected non-Alzheimer’s pathophysiology or primary age-related tauopathy. Yet its cognitive correlates are unclear, particularly when vascular neuropathologies coexist. We examined whether vascular neuropathologies are linked to cognitive impairment proximate to death and pre-mortem cognitive decline among decedents with intermediate-to‐high Braak stage (III-VI) and absent/low neuritic plaques.

**Methods:**

In 579 autopsied participants from the National Alzheimer’s Coordinating Center cohort (Braak III–VI; CERAD C0–C1), we evaluated arteriolosclerosis, atherosclerosis of the circle of Willis, cerebral amyloid angiopathy, gross infarcts/lacunes, and microinfarcts effect on harmonized memory, executive function, and language z-scores proximate to death using multivariable linear regression (adjusted for age, sex, education, APOE ε4 status). Linear mixed‐effects models assessed interactions between each vascular neuropathology and years‐to‐death on pre-mortem longitudinal decline.

**Results:**

At the last assessment, microinfarcts were associated with lower memory (β=–0.28, 95% CI − 0.51 - − 0.05; *p* = 0.02), executive function (β=–0.24, 95% CI − 0.44 - − 0.04; *p* = 0.02), and language (β=–0.21, 95% CI − 0.38 - − 0.04; *p* = 0.02). These associations remained after controlling for cardiovascular risk, neuritic plaques and Braak stage, last assessment and death interval, and co-existing vascular neuropathologies. Microinfarcts were not associated with the rates of pre-mortem cognitive decline.

**Conclusions:**

Microinfarcts are contributors to domain-specific cognitive deficits in tangle‐predominant, low‐amyloid older adults. These findings underscore a vascular‐neurodegenerative pathway distinct from classic Alzheimer’s disease. Thus, targeting microvascular injury may mitigate impairment in this underrecognized phenotype.

**Supplementary Information:**

The online version contains supplementary material available at 10.1186/s13195-025-01863-y.

## Introduction

Cognitive decline in late life is increasingly understood as the result of multiple overlapping pathologies (i.e., polypathology) that contribute to mixed dementia, despite the majority of individuals receiving a clinical diagnosis of Alzheimer’s disease (AD) [1–5]. Traditionally, AD was clinically defined by the accumulation of amyloid-β (Aβ) plaques (A+) and neurofibrillary tau tangles (T+). However, recent diagnostic frameworks have shifted toward a biological definition of AD, incorporating evidence of amyloid (A), neurofibrillary tau (T), and neurodegeneration (N) to better understand the clinical significance and interactions of distinct AD pathology [6, 7].

Historically, definitive AD diagnosis required autopsy with the identification of the density and spread of Aβ plaques and neurofibrillary tau tangles using immunohistochemical or special stains [8]. But with advances in positron emission tomography and cerebrospinal fluid biomarkers, and more recently, plasma-based biomarkers, the clinical diagnosis of AD has become more biologically accurate with technological advancements [6, 7]. Despite the central role of amyloid and tau in AD, not all individuals follow a stereotypical pattern of pathology. Some individuals demonstrate intermediate or advanced tau pathology in the absence of amyloid deposition, suggesting alternative mechanisms of neurodegeneration [9, 10]. Mounting evidence indicates that vascular neuropathologies accompany AD and Lewy body disease (LBD), including arteriolosclerosis, atherosclerosis, cerebral infarcts, and microvascular injury which can contribute to late-life cognitive decline and dementia both independent of and in consort with AD-related Aβ pathology [11, 12]. However, the distinct impact of vascular pathology on cognition, particularly in the context of predominantly neurofibrillary tau and none to low neuritic plaques, remains underexplored.

Large autopsy-based cohort studies have consistently shown that vascular lesions are highly prevalent in aging brains [12, 13]. Arteriolosclerosis and atherosclerosis are arguably the most prevalent vascular neuropathologies found at autopsy and also coexist with AD pathology [12, 14]. Vascular contributions to cognitive impairment appear to follow distinct mechanistic pathways compared to AD pathology and exerts effects even in individuals with minimal amyloid deposition. Studies have shown that vascular injury may precede or act independently of amyloid, serving as an early and potentially modifiable driver of neurodegeneration [15, 16]. For example, our recent findings demonstrate that arteriolosclerosis is associated with poorer cognitive outcomes independent of AD neuropathologic change (ADNC) and LBD pathology [12].

Microinfarcts (i.e., small ischemic lesions often undetected on neuroimaging) have been strongly associated with deficits in memory and executive function, despite adjusting for AD pathology [17]. Gross infarcts and large-vessel atherosclerosis are also robust predictors of dementia, especially among older adults with low or unclear AD biomarkers [18–20]. These findings provide further evidence that vascular pathology may not only interact with traditional neurodegenerative processes but may also independently mimic or drive cognitive impairment in the absence of amyloid pathology.

A growing number of older adults exhibit neurofibrillary tau pathology without amyloid plaques, a biomarker profile often referred to as primary age-related tauopathy (PART) in post-mortem studies or suspected non-Alzheimer’s pathophysiology (SNAP) clinically [9, 10]. The cognitive outcomes of this group are not well characterized, and vascular pathology may be a key determinant of symptom expression. Given the high prevalence of vascular disease in aging populations, and its well-established impact on white matter injury, cerebral perfusion, and inflammation, there is a pressing need to better understand vascular contributions to cognitive decline beyond traditional AD pathology accumulation. Doing so may reveal non-amyloid pathways of neurodegeneration and provide opportunities for intervention that have been overlooked in amyloid-centric models of dementia.

In this study, we leveraged harmonized demographic data, autopsied verified neuropathology collection, and harmonized cognitive scores from the National Alzheimer’s Coordinating Center (NACC) autopsy dataset to investigate the role of vascular neuropathologies (arteriolosclerosis, atherosclerosis of the circle of Willis, cerebral amyloid angiopathy, gross infarcts/ lacunes, and microinfarcts) in cognitive outcomes among individuals with intermediate or high neurofibrillary tau pathology but none or low neuritic plaques. We specifically investigated whether vascular neuropathologies significantly contribute to cognitive impairment at the last visit prior to within two years of death and pre-mortem cognitive decline across multiple cognitive domains (memory, executive function, language and global impairment) among neurofibrillary tangle predominant decedents.

## Methods

### National alzheimer’s coordinating center

In this study, the NACC neuropathology dataset represents a sub-sample from data acquired from the September 2024 [21]. The NACC serves as a central repository for standardized research data collected from Alzheimer’s Disease Research Centers (ADRC) across the United States. Funded by the National Institute on Aging, the ADRC began contributing data to NACC in 2005 through regular assessments of enrolled participants. Participants are enrolled across the full spectrum of cognitive function, from cognitively normal to those with mild cognitive impairment (MCI), non-MCI cognitive impairment, and dementia. All participating ADRC obtained approval from their respective institutional review boards prior to data collection. For the present study, the decedents included were from 34 ADRC. The autopsied data set generated from variable (NACCAUTP) yielded 8,188 participants.

### Data cleaning and processing

To prepare key neuropathological and genetic variables for analysis, several variables were recoded and cleaned in a harmonized autopsy-confirmed dataset. Apolipoprotein (*APOE*) ε4 carrier status was derived from the NACCAPOE variable, with participants coded as 1 if they carried at least one ε4 allele (e3/e4, e4/e4, or e2/e4) and 0 if they were non-carriers (e2/e2, e2/e3, e3/e3). This method has been similarly used on other NACC neuropathology research [14].

ADRC uploaded data to NACC using neuropathology forms 9 and 10 per consensus guidelines [8, 22]. Following previous NACC methodologies and aligning with clinical influence on cognition, the vascular neuropathological markers were categorized as binary: arteriolosclerosis (NACCARTE), atherosclerosis of the circle of Willis (henceforth atherosclerosis) (NACCAVAS), and cerebral amyloid angiopathy (CAA) (NACCAMY) were binarized to indicate presence (moderate or severe pathology) versus absent/mild [12, 23]. Additionally, the variables gross infarcts/lacunes (henceforth referred to as gross infarcts) (NACCINF) and microinfarcts (NACCMICR) were originally coded as binary variables [12, 23].

To quantify tau pathology, the Braak stage (NACCBRAA) was recoded into an ordered categorical variable. Braak stages were recategorized into four ordinal groups based on standard staging conventions: stage 0 (Braak = 0) was labeled as “B0” (no neurofibrillary pathology); stages 1–2 as “B1” (trans-entorhinal); stages 3–4 as “B2” (limbic); and stages 5–6 as “B3” (isocortical involvement). We used the neuritic plaque density variable (NACCNEUR), which was recorded in severity levels ranging from 0 to 3, to account for neuritic amyloid plaque pathology. We isolated participants with moderate-to-severe tau burden (Braak stage B2 or B3) and none/low neuritic plaque density (NACCNEUR score of 0 or 1) for our analytical sample. This combination reflects individuals with predominately tau-positive neurofibrillary tangle pathology in the context of none/ low neuritic plaques, which may represent an alternative neurodegenerative pathway that does not follow the traditional amyloid cascade hypothesis.

### Neuropathology exclusions in intermediate/high neurofibrillary Tau (T+) and low or no neuritic plaque (T + A-) cohort

To isolate a neuropathologically homogenous sample and reduce confounding from comorbid pathological processes, we applied sequential exclusion criteria to remove participants with non-AD pathologies aligned with previous studies [12, 24]. We first excluded individuals with vascular pathology (e.g., cerebral autosomal dominant arteriopathy with subcortical infarcts and leukoencephalopathy (CADASIL)) based on NPPVASC and NPPATH10 variables. We next excluded participants with frontotemporal lobar degeneration (FTLD), prion disease, tauopathies, and related proteinopathies by removing those with positive codes (value = 1) for a comprehensive set of neuropathological flags, including NPPFTLD, NPCFTLD, NPPPRION, NPCPRION, NPFTDTAU, NPFTDTDP, NPFTDNO, NPFTDSPC, NPFTDT2 through NPFTDT10, and NPOFTD through NPOFTD5. Similarly, cases positive for Pick’s disease (NACCPICK), CBD (NACCCBD), progressive supranuclear palsy (NACCPROG), and hippocampal sclerosis (NPPHIPP, NPCHIPP) were removed. We further excluded participants with amyotrophic lateral sclerosis motor neuron disease (NPALSMND), chromosomal abnormalities (NPCHROM, NACCDOWN), and a wide array of other rare or undefined neuropathologies (e.g., NPPDXA–NPPDXN, NPPOTH1–NPCOTH3, and NPTDPA–NPTDPE). This process yielded a final analytic cohort of 579 participants, reflecting a conservative sample with reduced neuropathological heterogeneity, aligned with our hypothesis.

### Harmonized cognitive domain score measurements

The harmonized cognitive domain scores based on neuropsychological assessment developed for NACC and other clinical and population based cohorts have been previously described [25]. In brief, a multidisciplinary panel reviewed cognitive test items and classified them into core cognitive domains, including memory, executive function, language, and visuospatial ability, while some items were not assigned to any domain. These classifications informed the use of confirmatory factor analysis conducted on a pooled dataset to establish latent cognitive constructs. Item response theory methods were applied to derive factor scores and standard errors using anchor items shared across studies. A visuospatial harmonized cognitive score was not derived for NACC since there was limited item data related to that document available at the NACC visits. The harmonized cognitive scores outcomes are z-scored ranges.

### NACC harmonized cardiovascular disease risk score

To standardize cardiovascular risk factor data across multiple cohorts, including NACC, a harmonized workflow was developed and is described elsewhere [26]. The harmonization workflow utilized participants’ self-reported history of heart disease, hypertension (HTN), and diabetes mellitus (DM). In addition, body mass index (BMI) from the most recent study visit was included. Reports of any cardiovascular condition, regardless of severity or timing, were considered sufficient for inclusion. The four indicators, heart disease, HTN, DM, and BMI, were integrated into a composite cardiovascular risk score using principal component analysis. The first principal component, which captured the greatest variance among the four indicators, was retained as the primary risk score. Full documentation of the harmonization process is available in the Cardiovascular Harmonization Workflow README file [27], with methodology further described by Lee et al. [27] and the Alzheimer’s Disease Sequencing Project Phenotype Harmonization Consortium (ADSP-PHC) [26].

## Statistical analysis

### Heterogeneity and correlation of neuropathologies

First, we visualized the heterogeneity in neuropathology combinations using an UpsetPlot. Second, to evaluate the distribution of co-occurring neuropathologies in the selective sample, we quantified the burden of comorbid pathologies among participants with Braak stage B2 and B3, respectively. As described, binary indicators for each neuropathology (neuritic plaques, arteriolosclerosis, atherosclerosis, CAA, gross infarcts, and microinfarcts) were used. Participants with Braak B2 (or B3) were isolated, and the corresponding Braak indicator was removed from the matrix to avoid artificial inflation of pathology count for stratification of the polypatholgy distribution. In the B2 group and B3 group, we separately computed a total count of co-pathologies by summing binary indicators across all remaining pathologies. We then summarized the frequency and percentage of participants exhibiting 0 to 6 co-pathologies and visualized these distributions. Third, to evaluate the interrelationships among neuropathologies in this selective sample, we computed pairwise Pearson correlation coefficients for binary pathology indicators, including Braak stage (B2 and B3 separately), neuritic plaques (C0 vs. C1), and the five vascular pathologies. Correlation coefficients were calculated using pairwise complete observations. To assess statistical significance, we conducted individual Pearson correlation tests and reported corresponding *p*-values. Heatmaps were generated to visualize the correlation structure, with cell color representing the magnitude and direction of Pearson *r* values and numeric labels overlaid for interpretability.

### Logistic regression

To investigate the relationship between vascular neuropathologies and clinical dementia status/cognitive impairment, we fit separate multivariable logistic regression models. The binary outcome variable, “any dementia,” was derived by recoding the Clinical Dementia Rating Global Score (CDR) where 0 indicated no dementia/ cognitive impairment and > 0 indicating the presence of cognitive impairment/dementia. Each of the five models examined a different vascular neuropathology (arteriolosclerosis, atherosclerosis, CAA, gross infarcts, and microinfarcts) as the primary predictor. All models were adjusted for age at death, sex, education, and *APOE* ε4 status. The base models included one vascular predictor and covariates. The fully adjusted model included all 5 vascular neuropathologies. Results are reported as odds ratios (OR) with corresponding 95% confidence intervals (CI) and p-values.

### Sensitivity analysis ordinal logistic regression

To evaluate associations between vascular neuropathology and CDR stages, we used ordinal logistic regression models with global CDR as the outcome. For modeling purposes, participants with global CDR scores of 0 or 0.5 were grouped together and coded as 0, representing the lowest level of impairment, while higher scores reflected increasing dementia severity (1 vs. 2 vs. 3). All models were adjusted for age at death, sex, education, and *APOE* ε4 status. The base models included one vascular predictor and covariates. The fully adjusted model included all 5 vascular neuropathologies. Results are reported as OR with 95% CI and p-values.

### Multivariable linear regression (MVLR)

We conducted domain-specific multivariable linear regression models to examine the cross-sectional associations between vascular neuropathologies and cognitive scores proximate to death. Outcomes included memory, executive function, and language. For each outcome, five separate models were estimated (one per vascular predictor) adjusting for age at death, sex, years of education, and *APOE* ε4 status. Results are reported as beta coefficients (β) with 95% CI.

### Sensitivity analysis (MVLR): adjustment for cardiovascular risk

To address potential confounding by systemic cardiovascular health, we repeated the above analyses with additional adjustment for a harmonized cardiovascular risk score derived from the ADSP-PHC [26]. Linear regression models were again stratified by cognitive outcome and included all prior described covariates with the cardiovascular risk score and included one vascular neuropathology.

### Sensitivity analysis (MVLR): neuritic plaques (C1)-negative subgroup

To isolate the impact of vascular neuropathology in the absence of neuritic plaques, we restricted analyses to participants with elevated neurofibrillary tau pathology (Braak stages B2 or B3) and no neuritic plaques (NACCNEUR = 0; removed participants with NACCNEUR = 1). Linear regression models were again stratified by cognitive outcome and included all prior described covariates and included one vascular neuropathology.

### Sensitivity analysis (MVLR): adjusting for Tau (Braak stage) and neuritic plaque pathological groups

To evaluate the independence of vascular effects from Alzheimer’s hallmark pathologies, we adjusted for both Braak stage (B2 vs. B3) and neuritic plaque burden (C0 vs. C1). Linear regression models were employed for each cognitive outcome and included all prior described covariates and included one vascular neuropathology.

### Sensitivity analysis (MVLR): adjusting for neuritic plaques and individual Braak staging

Individual regression models were fit for each vascular neuropathology and cognitive outcome, adjusting for age at death, sex, years of education, and APOE ε4 status. In sensitivity analyses, models were additionally adjusted for neuritic plaque burden (C0 vs. C1) and Braak stage, modeled as a categorical variable (Braak III, IV, V, VI).

### Sensitivity analysis (MVLR): adjusting for years between last exam and death

Another sensitivity analysis was run adjusting for the time in years between the last clinical exam and the participant’s death in regression models for each vascular neuropathology and cognitive outcome, adjusting for age at death, sex, years of education, and APOE ε4 status.

### Sensitivity analysis (MVLR): adjusting for all 5 vascular neuropathologies (Independent associations)

To assess vascular predictor independence, we included regression models included for all 5 vascular neuropathologies adjusting for age at death, sex, years of education, and APOE ε4 status for each cognitive outcome.

### Sensitivity analysis (MVLR): adjusting for all 5 vascular neuropathologies (Independent associations) and years between last exam and death

In the models that included all 5 vascular predictors and covariates, we also adjusted for the time in years between the last clinical exam and the participant’s death.

### Linear mixed-effects models (LMEM)

We used linear mixed-effects models (LMEM) to evaluate the associations between neuropathologic predictors and longitudinal cognitive domain decline prior to death. In primary models, we tested NACCNEUR and Braak stage (B2 vs. B3); in secondary models, we examined each of five vascular neuropathologies. All models included fixed effects for years to death (time), neuropathology status (e.g., CERAD 0 vs. 1; pathology absent vs. present), and their interaction, adjusting for sex, years of education, and APOE ε4 status. Random intercepts and slopes for time were included to account for within-person variability. Models were fit using maximum likelihood estimation. Interaction terms were probed using marginal effects plots, with β coefficients representing differences in cognitive domain decline rate by neuropathology status, reported with 95% CI.

Model example: (cognitive domain ~ years prior to death * neuropathologic predictor + sex + education + APOE e4 + (years prior to death | participant identifier).

### Linear mixed-effects models (LMEM) sensitivity analyses

To assess the robustness of our findings, we conducted sensitivity analyses excluding participants with any evidence of neuritic plaques (CERAD = 1). We refit the LMEM for each vascular neuropathology to examine whether our significant and null findings were consistent with longitudinal cognitive domain decline in the absence of neuritic plaque burden.

All statistical analyses were conducted using R version 4.2.3. All p-values are two-sided, and the statistical significance was set at p-value < 0.05 for all demographic comparisons. Based on our a priori hypotheses, we did not perform multiple comparisons which should be considered when interpreting the findings from the p-values versus the 95% CI.

### Data availability

All data used in the current analyses is available for download from the NACC (https://www.alz.washington.edu/*).*

## Results

### Sample characteristics

Among the 579 decedents with none/low neuritic plaques and intermediate/high neurofibrillary tau pathology, the majority were White (95.7%), with a mean age at death of 88.4 years and nearly half identified as male (49.4%) (Table 1). Additionally, 29.5% were *APOE* ε4 carriers. The majority of the sample had a CERAD of 1 (C1) (59.9%) and was classified at Braak stage B2 (82.4%). Vascular neuropathologies were prevalent with atherosclerosis (43.2%) and arteriolosclerosis (40.2%) being the most common. In terms of cognitive status, 30.9% had no dementia/cognitive impairment (CDR = 0). The primary etiologic diagnosis in this analytical sample was AD (39%), despite lacking intermediate and advanced neuritic plaques pathology (Supplemental Table 1). An additional 32% were classified as clinically cognitively normal at their visit most approximate to death, suggesting substantial clinical heterogeneity within this subgroup. Vascular dementia or vascular brain injury accounted for 6% of diagnoses, while neurodegenerative conditions such as LBD (13.5%) and various non-AD pathologies were also represented in small percentages.

**Table 1:** Demographics, neuropathology, and clinical characteristics of neurofibrillary tangle predominant decedents

	Overall (*N* = 579)
Age at Death, years Mean (SD)	88.4 (8.6)
Male, n (%)	286 (49.4)
Education, years Mean (SD)	15.6 (3.1)
Missing, n (%)	5 (0.9)
Hispanic Ethnicity, n (%)	14 (2.4)
Missing/Unknown, n (%)	1 (0.2)
Race, n (%)	
White	554 (95.7)
Black or African American	18 (3.1)
American Indian or Alaska Native	1 (0.2)
Native Hawaiian or Other Pacific Islander	0 (0)
Asian	2 (0.3)
Unknown	2 (0.3)
*APOE* ε4 carrier status, n (%)	171 (29.5)
Missing, n (%)	49 (8.5)
Neuritic Plaques	
CERAD 0, n (%)	232 (40.1)
CERAD 1, n (%)	347 (59.9)
Braak Stage	
B2, n (%)	477 (82.4)
B3, n (%)	102 (17.6)
Arteriolosclerosis, n (%)	233 (40.2)
Missing, n (%)	58 (10)
Atherosclerosis of the circle of Willis, n (%)	250 (43.2)
Missing, n (%)	6 (1)
CAA, n (%)	104 (18.0)
Missing, n (%)	6 (1)
Gross Infarcts/ Lacunes, n (%)	115 (19.9)
Missing, n (%)	4 (0.7)
Microinfarcts, n (%)	163 (28.2)
Missing, n (%)	1 (0.2)
Memory Mean (SD)	-0.1 (1.1)
Missing, n (%)	176 (30.4)
Executive Function Mean (SD)	-0.1 (0.8)
Missing, n (%)	265 (45.8)
Language Mean (SD)	-0.1 (0.8)
Missing, n (%)	200 (34.5)
CDR Global, n (%)	
0	179 (30.9)
0.5	156 (26.9)
1	84 (14.5)
2	77 (13.3)
3	83 (14.3)

This table presents the demographic, neuropathological, and cognitive characteristics of 579 decedents with none/low neuritic amyloid plaques and intermediate/high tau pathology. Variables include age at death, sex, years of education, ethnicity, and race. Neuropathological measures include CERAD neuritic amyloid plaque scores, Braak staging for tau pathology, arteriolosclerosis, atherosclerosis, cerebral amyloid angiopathy (CAA), microinfarcts, gross infarcts/lacunes, and *APOE* (apolipoprotein) ε4 carrier status. Cognitive assessments include domain-specific scores for memory, executive function, and language, as well as global cognitive status using the Clinical Dementia Rating (CDR) scale. Data are reported as means with standard deviations (SD) for continuous variables and counts with percentages for categorical variables. Missing data are noted where applicable

### Heterogeneity of the sample

**Fig. 1:**
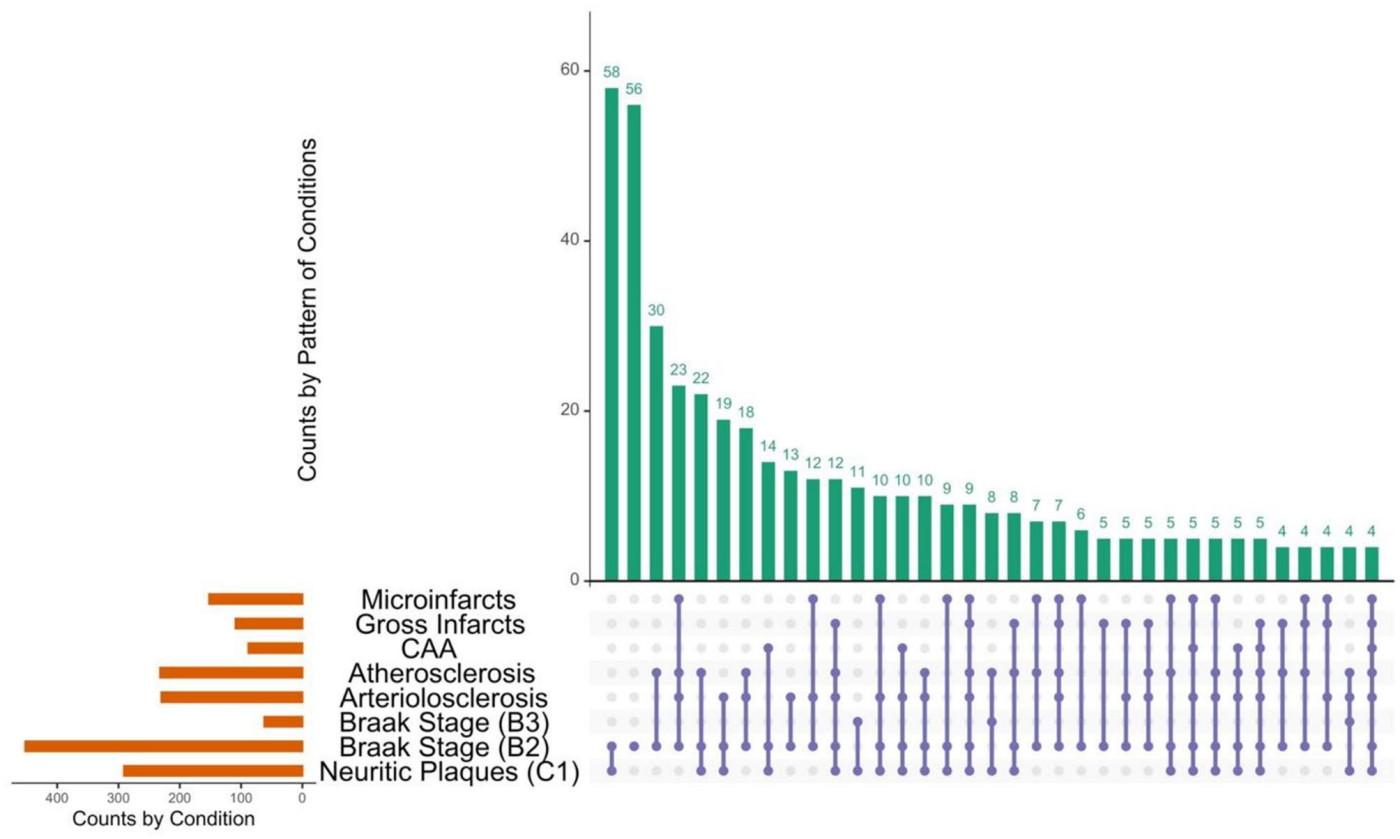
Prevalence and co-occurrence of neuropathologies in the selected sample. The left horizontal bar chart shows the marginal frequency of each neuropathology in the cross-sectional sample (orange bars): neuritic plaques, Braak neurofibrillary tangle stages II (B2) and III (B3), arteriolosclerosis, atherosclerosis, cerebral amyloid angiopathy (CAA), gross infarcts, and microinfarcts. The right panel is an UpSet plot of the intersection frequencies (teal bars): each bar’s height is the number of participants exhibiting exactly that combination of pathologies. In the matrix below, filled purple circles denote presence of that neuropathology in the combination, light-gray circles denote absence, and connecting lines group the circles into the same intersection set. The UpSet plot was limited for visibility purposes.

Copathology or polypathology was extremely common in this sample (Fig. 1; Supplemental Table 2). Neuritic plaques and Braak stage B2 (*n* = 58) and only B2 (*n* = 56) were the two most prevalent conditions at death. Some notable copathology patterns included B2 with atherosclerosis (*n* = 30), B2 with arteriolosclerosis, atherosclerosis, and microinfarcts (*n* = 23), and neuritic plaques with B2, and atherosclerosis (*n* = 22). By contrast, polypathology was more prevalent in the sample than having a single neuropathology.

### Comorbid neuropathologies stratified by Braak B2 and B3

**Fig. 2:**
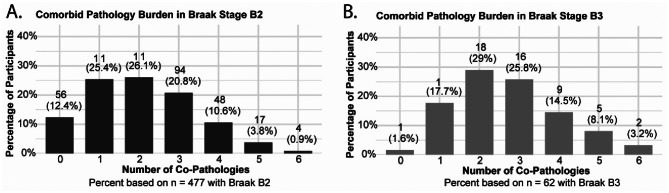
Distribution of comorbid neuropathologic counts among decedents stratified by Braak stage (B2 versus B3). Co-pathologies were defined as the number of additional neuropathologic lesions (neuritic plaques, arteriolosclerosis, atherosclerosis, cerebral amyloid angiopathy, microinfarcts, and gross infarcts) present alongside neurofibrillary tangle burden. Counts and percentages reflect the number of participants with zero through six co-pathologies within each Braak stage subgroup (B2: *n* = 477; B3: *n* = 62)

In participants with Braak B2 neurofibrillary tangles (*n* = 477), 25% exhibited exactly one other neuropathology (*n* = 115, 25.4%) and 25% exhibited two neuropathologies (*n* = 124, 26.1%) (Fig. 2A; Supplemental Table 2). Only 12.4% (*n* = 56) had B2 level of tangles while the remainder of the sample presented with three or more neuropathologies. A similar trend was observed in decedents with more advanced neurofibrillary tau (B3) (Fig. 2B; Supplemental Table 2).

### Correlation of neuropathologies

**Fig. 3:**
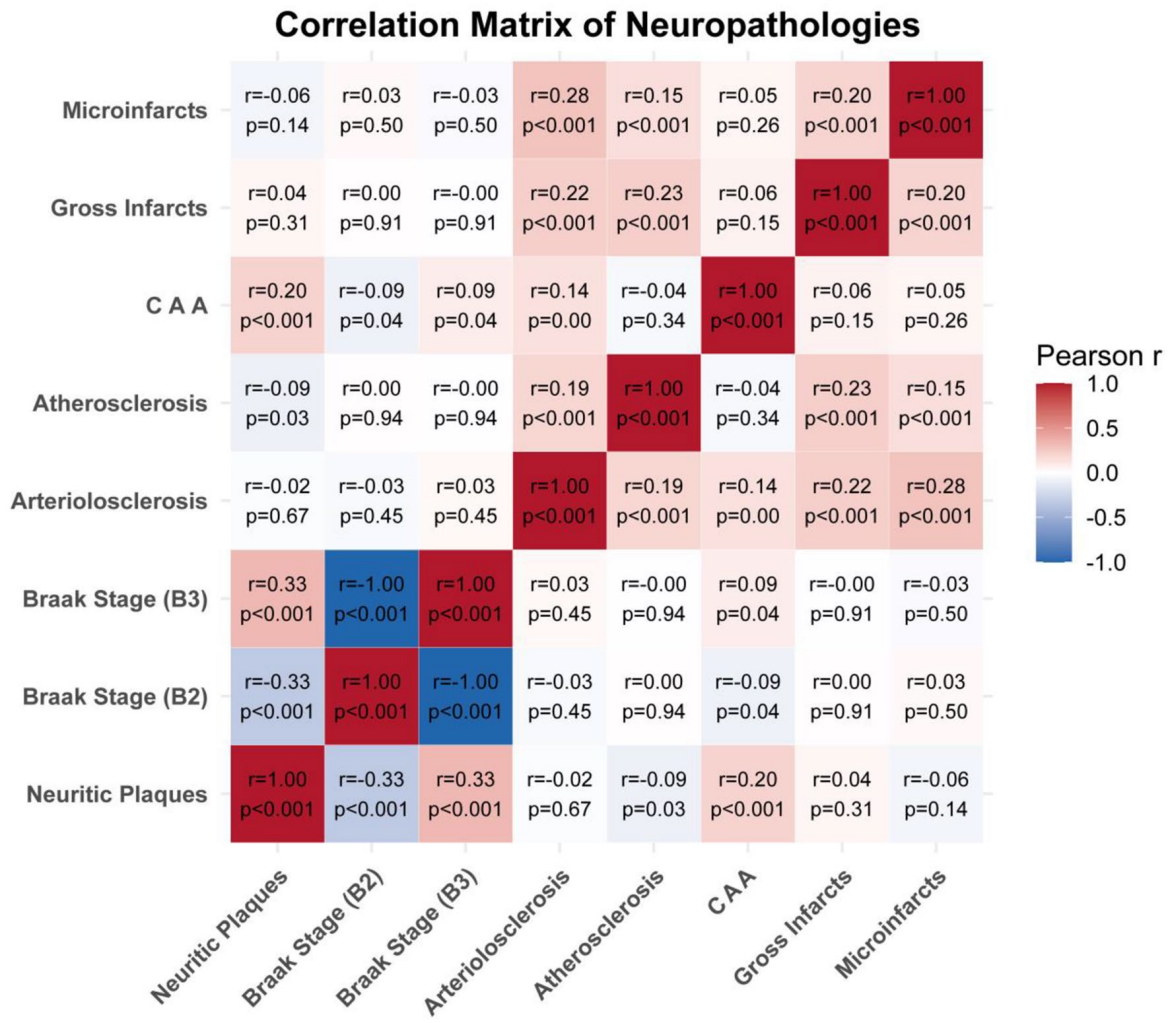
Correlation matrix of neuropathologies. Heatmap of pairwise Pearson correlations (r) among eight binary-coded neuropathologic lesions: neuritic plaques, Braak stage II (B2) and stage III (B3) tangles, arteriolosclerosis, atherosclerosis, cerebral amyloid angiopathy (CAA), gross infarcts, and microinfarcts. Cell color ranges from deep blue (*r* = –1.0) through white (*r* = 0) to deep red (r = +1.0). Overlaid text gives the exact r and its two‐sided p-value. Negative r values indicate inverse correlations ; positive r values indicate positive correlations

Pairwise correlations among neuropathologies were generally weak to modest (|r| < 0.3) (Fig. 3; Supplemental Table 3). The strongest positive association was between arteriolosclerosis and microinfarcts (*r* = 0.28, *p* < 0.001), followed by arteriolosclerosis–atherosclerosis (*r* = 0.19, *p* < 0.001) and microinfarcts–atherosclerosis (*r* = 0.15, *p* < 0.001). Neuritic plaques were positively correlated with CAA (*r* = 0.20, *p* < 0.001). Gross infarcts exhibited only weak correlations with other markers (all |r| ≤ 0.23, *p* > 0.05 for several comparisons).

### Association between vascular neuropathology and clinical dementia rating

In minimally adjusted logistic models (age at death, education, sex, and *APOE* ε4 carrier status), arteriolosclerosis was associated with 72% higher odds of any dementia (global CDR > 0; OR = 1.72, 95% CI 1.15–2.57; *p* = 0.01), atherosclerosis with 61% higher odds (OR = 1.61, 95% CI 1.07–2.43; *p* = 0.02), gross infarcts with an almost four-fold higher odds (OR = 3.98, 95% CI 2.29–7.30; *p* < 0.01), and microinfarcts with nearly doubled odds (OR = 1.95, 95% CI 1.26–3.08; *p* < 0.01) (Table 2). CAA was not significantly related to dementia (OR = 1.12, 95% CI 0.67–1.92; *p* = 0.66) (Table 2). After mutual adjustment for all five vascular lesions plus the same covariates, only gross infarcts remained a significant predictor (OR = 3.32, 95% CI 1.87–6.19; *p* < 0.01). In ordinal logistic models adjusting for the same covariates, only gross infarcts were significantly associated with greater dementia severity (base model OR = 2.02, 95% CI 1.32–3.10; *p* < 0.01), and when all five vascular lesions were entered simultaneously (fully adjusted model), the effect of gross infarcts remained (OR = 1.86, 95% CI 1.16–2.97; *p* = 0.01) (Supplemental Table 4).

**Table 2:** Association between vascular neuropathologies and global clinical dementia rating greater than zero

Predictor	OR (95% CI), Base Model	OR (95% CI), Fully Adjusted
Arteriolosclerosis	**1.72 (1.15–2.57)**,** 0.01**	1.31 (0.85–2.03), 0.22
Atherosclerosis	**1.61 (1.07–2.43)**,** 0.02**	1.18 (0.76–1.84), 0.45
CAA	1.17 (0.69–2.01), 0.57	0.92 (0.52–1.63), 0.76
Gross Infarcts	**3.98 (2.29–7.30)**,** < 0.01**	**3.32 (1.87–6.19)**,** < 0.01**
Microinfarcts	**1.95 (1.26–3.08)**,** < 0.01**	1.56 (0.97–2.54), 0.07

This table displays the odds ratios (OR) with 95% confidence intervals (CI) and corresponding p-values for the association between vascular neuropathologies and the presence of any dementia defined as a global Clinical Dementia Rating (CDR) greater than 0 using logistic regression. Vascular neuropathologies examined include arteriolosclerosis, atherosclerosis, cerebral amyloid angiopathy (CAA), gross infarcts, and microinfarcts. The base models controlled for age at death (years), years of education, sex, and apolipoprotein ε4 carrier status. The fully adjusted models included all 5 vascular neuropathologies and the covariates. P-values < 0.05 were considered significant

### Cross-sectional associations between vascular neuropathologies and harmonized cognitive scores

At the visit proximate to death, microinfarcts were significantly associated with poorer memory (*β* =-0.27, 95% CI: -0.50 – -0.05, *p* = 0.02), executive function (*β* =-0.24, 95% CI: -0.44 – -0.04, *p* = 0.02), and language performance (*β* =-0.21, 95% CI: -0.37 – -0.04, *p* = 0.02, Table 3) while no other vascular neuropathologies were associated with any cognitive scores. In sensitivity analyses when controlling for harmonized cardiovascular disease risk, microinfarcts remained associated with lower harmonized cognition across all domains (Supplemental Table 5). In a sample without low neuritic plaques, microinfarcts were similarly associated with poorer memory and language scores but the effect was stronger for executive function (*β* =-0.48, 95% CI: -0.77 – -0.19, *p* < 0.01) (Supplemental Table 6). Gross infarcts were also associated with poorer memory performance in those without low neuritic plaques (*β* =-0.38, 95% CI: -0.75 – -0.01, *p* = 0.04) (Supplemental Table 6). The main effects of microinfarcts were similar as the primary findings when controlling for neuritic plaque levels and Braak staging (Supplemental Table 7). Results for microinfarcts on each cognitive domain were similar when adjusting for neuritic plaques and individual Braak stage (III (reference), IV, V, VI) (Supplemental Table 8) and when we added the time interval in years between the last cognitive exam and death (Supplemental Table 9). The effects of microinfarcts were also independent from the other four vascular neuropathologies as evidenced by the sensitivity analysis that included all vascular neuropathologies (Supplemental Table 10) and when controlling for the time between the last visit and death (Supplemental Table 11).

**Table 3:** Association between vascular neuropathologies and cross-sectional cognitive domain and dementia scores proximal to death

Predictor	Memory β (95% CI), *p*	Executive function β (95% CI), *p*	Language β (95% CI), *p*
Arteriolosclerosis	–0.12 (–0.33–0.09), 0.27	–0.11 (–0.30–0.07), 0.22	–0.14 (–0.28–0.01), 0.07
Atherosclerosis	–0.10 (–0.31–0.10), 0.31	–0.03 (–0.22–0.15), 0.73	0.07 (–0.09–0.23), 0.38
CAA	–0.16 (–0.44–0.12), 0.26	–0.06 (–0.30–0.18), 0.61	0.03 (–0.18–0.23), 0.80
Gross Infarcts	–0.23 (–0.49–0.04), 0.09	–0.08 (–0.31–0.15), 0.50	–0.18 (–0.38–0.02), 0.08
Microinfarcts	–0.27 (–0.50 – − 0.05), 0.02	–0.24 (–0.44 – − 0.04), 0.02	–0.21 (–0.37 – − 0.04), 0.02

Linear regression analyses examining the associations between vascular neuropathologies and cognitive domain scores (memory, executive function, and language) proximate to death. The table reports beta coefficients (β) with 95% confidence intervals (CI) and corresponding p-values for each vascular neuropathology, including arteriolosclerosis, atherosclerosis, cerebral amyloid angiopathy (CAA), gross infarcts, and microinfarcts. Negative β values indicate worse cognitive performance associated with the presence of the neuropathology. Statistical significance was defined as *p* < 0.05

### Effects of neuritic plaques and Braak staging on longitudinal cognitive decline prior to death

**Fig. 4:**
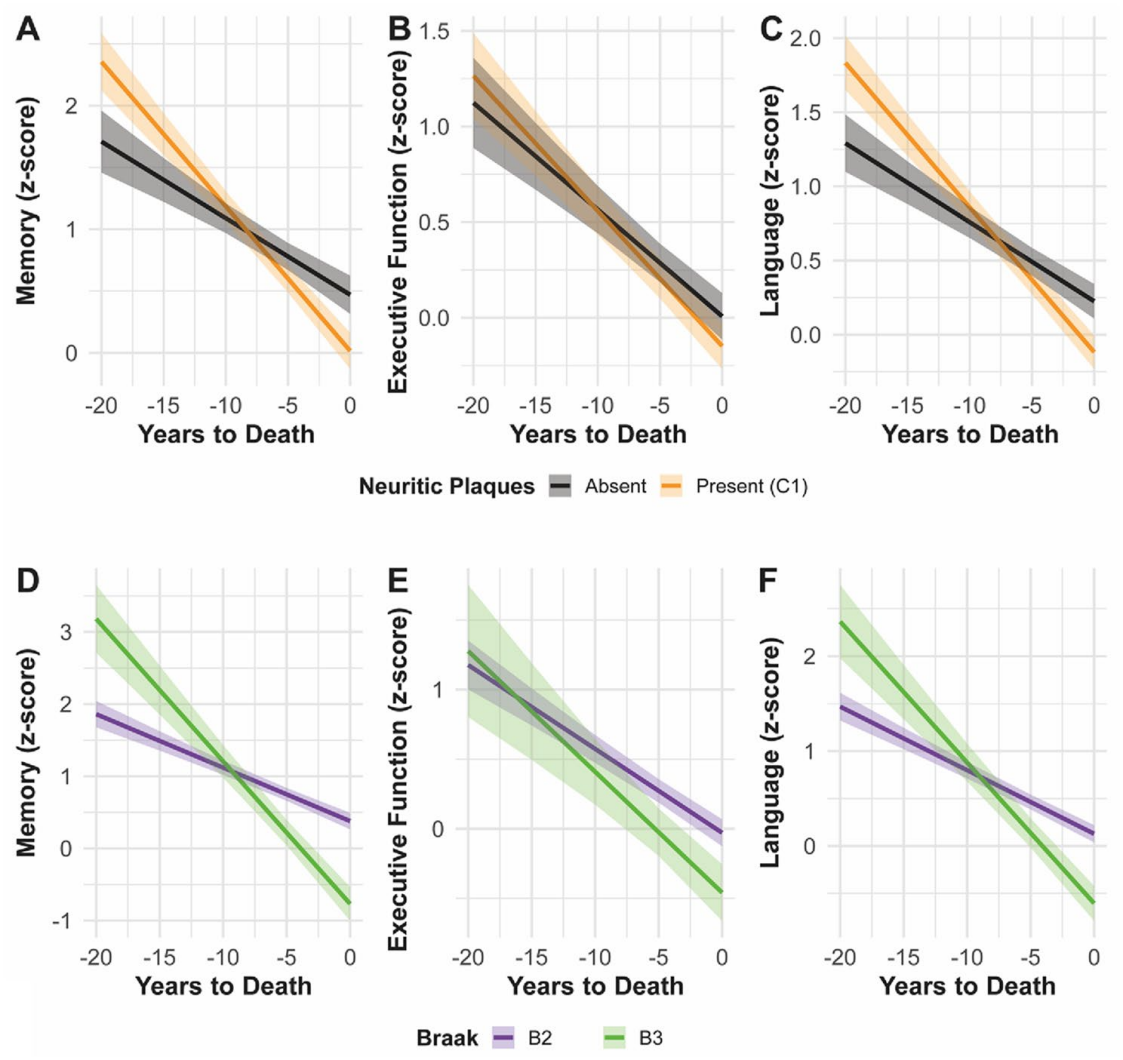
Predicted cognitive trajectories by neuritic plaques and Braak stage. Panels **A**–**C** depict model-predicted z‐scores for Memory (**A**), Executive Function (**B**), and Language (**C**) over the 20 years preceding death, stratified by neuritic plaque burden (black = absent; gold = present). Panels **D**–**F** show the same domains for Braak tangle stages (purple = stage II; green = stage III). All trajectories derive from linear mixed‐effects models with years‐to‐death × pathology interactions, holding sex, education, and APOE ε4 status at their reference/mean values. Shaded ribbons are 95% confidence intervals

In Model 1 (years-to‐death × neuritic plaques; Fig. 4A–C), the presence of neuritic plaques was associated with significantly steeper decline in memory (β = −0.05, 95% CI − 0.08 to − 0.03; *p* < 0.01) and language (β = −0.04, 95% CI − 0.06 to − 0.03; *p* < 0.01), but not in executive function (Table 4). Visually, the plaque‐present trajectory (gold line) diverges from the plaque‐absent trajectory (blak line) most markedly for memory and language (Fig. 4A ad C). In Model 2 (years‐to‐death × Braak stage III; Fig. 4D–F), more advanced tangle pathology (B3 vs. B2) similarly predicted faster memory loss (β = −0.12, 95% CI − 0.16 to − 0.09; *p* < 0.01) and language decline (β = −0.08, 95% CI − 0.11 to − 0.06; *p* < 0.01) (Table 4). Similar longitudinal effects were observed in the models including both neuropathology interactions (Table 4).

**Table 4:** Interaction effects of neuritic plaques and Braak stage on pre-mortem cognitive decline

Model	Predictors	Memory β (95% CI), *p*	Executive function β (95% CI), *p*	Language β (95% CI), *p*
Model 1	Years to death × Neuritic plaques (C1)	**–0.05 (–0.08 – − 0.03), < 0.01**	–0.01 (–0.03–0.00), 0.13	**–0.04 (–0.06 – − 0.03), < 0.01**
Model 2	Years to death × Braak (B3)	**–0.12 (–0.16 – − 0.09), < 0.01**	–0.03 (–0.06–0.00), 0.08	**–0.08 (–0.11 – − 0.06), < 0.01**
Model 3	Years to death × Neuritic plaques (C1)	**–0.03 (–0.05 – − 0.01), 0.01**	–0.01 (–0.03–0.01), 0.32	**–0.03 (–0.05 – − 0.01), < 0.01**
	Years to death × Braak (B3)	**–0.11 (–0.14 – − 0.08), < 0.01**	–0.02 (–0.05–0.01), 0.17	**–0.07 (–0.09 – − 0.04), < 0.01**

Linear mixed-effects models were used to examine the interaction between time to death and neuropathological markers on longitudinal cognitive outcomes across three cognitive domains. Model 1 includes the interaction term between years to death and neuritic plaque burden. Model 2 includes the interaction between years to death and Braak stage. Model 3 includes both interaction terms simultaneously. Estimates (β) reflect the slope difference in cognitive change over time associated with each neuropathology group. Confidence intervals (CI) are shown in parentheses. Statistically significant effects (*p* < 0.05) are bolded

### Effects of vascular neuropathologies on longitudinal cognitive decline prior to death

**Fig. 5:**
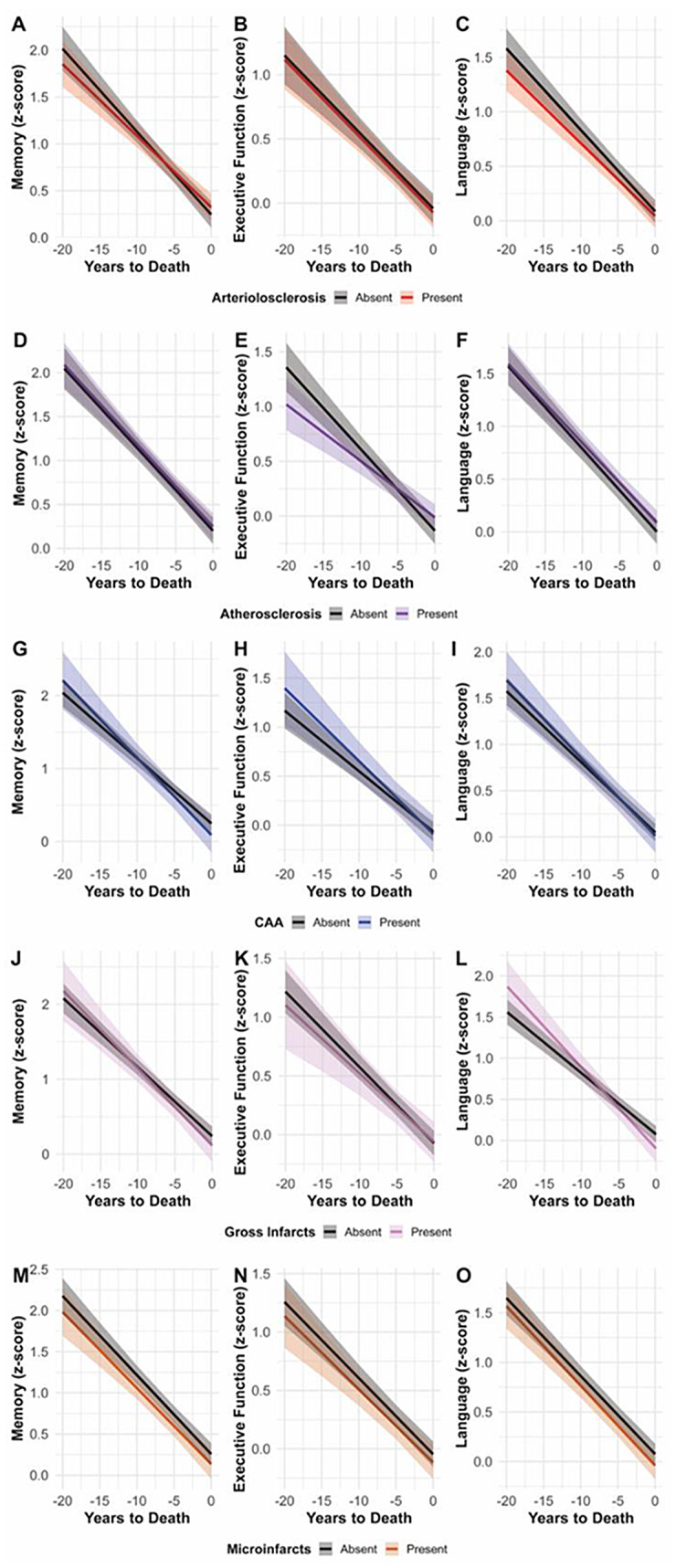
Pre-mortem harmonized cognitive domain trajectories by vascular neuropathology status. Linear mixed-effects model predictions of Memory (left), Executive Function (center), and Language (right) z-scores over the 20 years preceding death, stratified by presence (colored line) versus absence (black line) of each vascular neuropathology. Shaded ribbons = 95% confidence intervals. Panels **A–C**: Arteriolosclerosis (red = present). Panels **D–F**: Atherosclerosis (purple = present). Panels **G–I**: Cerebral Amyloid Angiopathy (CAA; blue = present). **Panels J–L**: Gross Infarcts (pink = present). Panels **M–O**: Microinfarcts (orange = present). All trajectories derive from linear mixed‐effects models with years‐to‐death × vascular neuropathology interactions, holding sex, education, and APOE ε4 status at their reference/mean values. Shaded ribbons are 95% confidence intervals

When evaluating whether vascular neuropathology modify pre-mortem slopes of cognitive decline (Fig. 5A-O; Table 5), only two domain‐specific effects reached statistical significance. The presence of atherosclerosis was associated with a slower cognitive decline in the executive‐function domain (years‐to‐death × atherosclerosis β = 0.02, 95% CI 0.00–0.04; *p* = 0.02) compared to decedents without. In contrast, gross infarcts were associated with a steeper longitudinal decline in language (years‐to‐death × gross infarcts β = − 0.02, 95% CI − 0.05 to − 0.00; *p* = 0.02) (Fig. 5L; Table 5). No significant interactions were observed for arteriolosclerosis, CAA, or microinfarcts in memory, executive function, or language (Fig. 5A-O; Table 5). None of the five vascular neuropathologies were associated with cognitive decline prior to death in those without neuritic plaques (Supplemental Table 12).

**Table 5:** Interaction effects between vascular neuropathologies and years to death on pre-mortem longitudinal cognitive outcomes

Predictors	Memory β (95% CI), *p*	Executive Function β (95% CI), *p*	Language β (95% CI), *p*
Years to death × Arteriolosclerosis	0.01 (–0.01–0.03), 0.29	0.00 (–0.02–0.02), 0.99	0.01 (–0.01–0.02), 0.32
Years to death × Atherosclerosis	0.00 (–0.02–0.02), 0.98	**0.02 (0.00–0.04), 0.02**	0.00 (–0.01–0.02), 0.69
Years to death × CAA	–0.02 (–0.05–0.01), 0.28	–0.01 (–0.04–0.01), 0.32	–0.01 (–0.03–0.01), 0.48
Years to death × Gross Infarcts	–0.01 (–0.04–0.02), 0.45	0.01 (–0.02–0.03), 0.62	**–0.02 (–0.05 – − 0.00), 0.02**
Years to death × Microinfarcts	0.00 (–0.02–0.03), 0.77	0.00 (–0.02–0.02), 0.81	–0.00 (–0.02–0.02), 0.86

Linear mixed-effects models were used to evaluate the interaction between time to death and each five vascular neuropathologies (arteriolosclerosis, atherosclerosis, cerebral amyloid angiopathy (CAA), gross infarcts, and microinfarcts) on cognitive trajectories. Outcomes included memory, executive function, and language. Interaction terms (years to death × vascular neuropathology) reflect differences in the rate of cognitive decline associated with each neuropathological marker. Estimates reflect the slope difference in cognitive change over time associated with each pathology group. Beta coefficients (β), 95% confidence intervals (CI), and p-values are reported for each domain. Significant associations (*p* < 0.05) are bolded

## Discussion

In this large, autopsy-confirmed cohort selected for intermediate‐to‐high neurofibrillary tangle burden with minimal neuritic plaques, we observed heterogeneity of comorbid pathologies: more than 80% of participants harbored two or more neuropathologies at death, and pure tangle or sparse plaque presentations were relatively uncommon. Cross‐sectionally, microinfarcts showed the strongest and most consistent associations with poorer performance across memory, executive function, and language proximate to death, even after adjusting for demographic factors, cardiovascular risk, plaque burden, Braak stage, and other vascular pathologies. Longitudinal mixed‐effects models revealed that both neuritic plaques and Braak stage (B3 compared to B2) independently accelerated pre-mortem decline in the memory and language domains. Arteriolosclerosis, CAA, and microinfarcts were not associated with premortem cognitive decline; however, we did observe a modest slowing of executive-function loss for those with atherosclerosis and steeper language decline for those with gross infarcts. Altogether, these findings highlight microinfarcts as a key vascular driver of end-stage impairment in tangle-predominant aging, while underscoring the primary role of amyloid and tau pathologies in shaping pre-mortem trajectories of decline.

This pathology profile has been understudied, with only one other study thoroughly investigating the role of vascular burden on A-T + or A-N + patients [28]. The increasing evidence of a tangle-predominant or tangle-only dementia has led to the acceptance of PART as a distinct entity apart from AD [9]. While our analyzed cohort is defined by no-to-sparse neuritic plaques and does not account for diffuse amyloid plaques due to coding and sample size constraints, our cohort best resembles those used in studies focused on PART [9]. Indeed, 30.9% of our cohort did not display cognitive impairment clinically (as defined by global CDR) which is in line with past autopsy studies reporting no significant antemortem cognitive impairment in individuals with PART or Aβ-independent tauopathy [29–31]. Our study particularly complements Besser et al. 2017 findings that increasing Braak stage and history of stroke was associated with cognitive impairment in individuals with neurofibrillary tangles and sparse neuritic plaques. Our study goes further by highlighting the robust association of microinfarctions to poorer memory, executive function, and language scores but had no effect on pre-mortem longitudinal cognitive decline. Importantly, the deleterious effects of microinfarcts on all three cognitive domains proximate to death remained when we controlled for all other vascular neuropathologies emphasizing the independence. Our study overall supports that vascular neuropathology, particularly microinfarcts, play a central role in cognitive impairment in the context of Aβ-independent tauopathy.

Importantly, the discrepancy between cross-sectional and longitudinal associations may reflect the cumulative nature of microinfarct-related injury, which is more detectable at a single time point when the burden has already manifested functionally [32–34]. Longitudinal models may be less sensitive to subtle or nonlinear effects of vascular pathology on decline, particularly when coexisting neuropathologies and cognitive reserve play modifying roles. Moreover, the inability to thoroughly detect microinfarcts in vivo limits our capacity to track their progression relative to cognitive change.

The deleterious effects of comorbid vascular neuropathologies including gross infarcts, and microinfarcts, on cognition independent of and in association with other neurodegenerative pathologies are well established [12, 35–37]. Of note, an autopsy study of individuals with AD and individuals with Aβ-independent tauopathy (i.e., PART) report that while the incidence of cerebrovascular disease and hemorrhages are greater in their AD group, both groups have similar frequency of gross infarcts (microinfarcts were not evaluated) [31]. Both groups in their study also experienced significant cognitive impairment in the presence of cerebrovascular pathology. Similarly, in our analysis, gross infarcts were linked to higher dementia severity and worse language function.

The more robust associations observed for microinfarcts may reflect their diffuse distribution and cumulative impact on cognitive networks. Microinfarcts are highly prevalent at autopsy, especially in older adults, and have been shown to contribute to dementia risk independent of concurrent Alzheimer’s pathology [32, 38, 39]. Their small size allows them to accumulate widely across cortex and subcortex, impairing connectivity and white matter integrity in ways that more focal lesions cannot capture [32]. In contrast, gross infarcts, although visually prominent on both imaging and pathology, are generally less prevalent in community-based samples and their cognitive consequences are highly variable depending on lesion location and size; after adjustment for coexisting pathologies, the overall effect of gross infarcts on cognition may be attenuated relative to microinfarcts [12].

Consistent with recent findings, Agrawal et al. demonstrated that cognitive decline in PART is more strongly associated with inferior temporal lobe tau deposition than hippocampal involvement, and that coexisting LATE-NC and vasculopathies (e.g., atherosclerosis, infarcts) exacerbate decline [40]. In our cohort characterized by transentorhinal, limbic, and isocortical tau involvement, it is plausible that some participants had variable hippocampal versus inferior temporal tau distribution and unrecognized LATE-NC, which, in combination with cerebrovascular disease, could have contributed to greater impairment. Our findings align with these additive effects, as arteriosclerosis, atherosclerosis, gross infarcts, and microinfarcts were associated with higher odds of a global CDR > 0, with microinfarcts in particular linked to poorer memory, executive function, and language. Similarly, another study reported that A–T + individuals—our focus group—had higher cerebrovascular burden (hypertension, diabetes, vascular lesions), although this difference was not statistically significant [41]. Notably, A–T + cases were significantly more likely than A + T– cases to receive a cognitive impairment/dementia diagnosis yet still performed better than A + T + cases. Additionally, in a study of Black decedents that included individuals with AD, LATE-NC, and Lewy body proteinopathies, the authors found that after controlling for these neurodegenerative conditions, microinfarcts in any region and basal ganglia arteriolosclerosis remained associated with lower global cognition, and mixed cerebrovascular pathologies were linked to poorer episodic memory and perceptual speed [42]. These results suggest that cerebrovascular disease may impact cognition independently of specific neurodegenerative diagnoses. This supports our conclusion that it contributes to cognitive decline in A–T + individuals, while also raising the possibility that unmeasured LATE-NC could have further influenced these outcomes.

### Clinical significance and importance

These findings indicate that evaluation and management of late-life cognitive impairment must extend beyond amyloid. In individuals classified as SNAP or PART, vascular co-pathology can substantially impair cognition even with low neurofibrillary tau, within the updated biological definition of AD [7, 43]. Plasma and neuroimaging biomarkers of microvascular injury and tau not contingent on amyloid positivity are therefore critical for risk stratification [43]. Results also support vascular-targeted prevention and treatment (e.g., antihypertensive, antithrombotic, anti-inflammatory strategies) regardless of amyloid status. Notably, ≥ 2 microinfarcts confer higher dementia risk even in the absence of AD pathology, underscoring the need to prioritize vascular health [44]. Finally, we advocate broader inclusion of biologically heterogeneous participants in studies and trials. Amyloid-negative/tau-positive individuals remain at meaningful risk via non-AD pathways; excluding them risks missed opportunities for tailored intervention. Embedding vascular assessments in diagnostic frameworks and eligibility criteria will better capture the full spectrum of late-life cognitive impairment and improve the real-world generalizability of therapies.

### Limitations and strengths

This cross-sectional design limits causal inference between vascular neuropathology and cognitive outcomes; longitudinal studies are needed to establish temporal relationships, particularly for microinfarcts. Site-level differences in NACC data collection and incomplete biomarker availability may contribute to measurement variability. Binary coding of vascular lesions may obscure dose–response effects; semi-quantitative staging could improve detection of clinically relevant vascular burden. Residual confounding from unmeasured factors is possible, and the predominantly non-Hispanic White sample limits generalizability to populations disproportionately affected by vascular disease and cognitive decline [45]. Strengths include the use of a large, harmonized autopsy dataset with detailed neuropathologic and cognitive measures, enabling targeted analysis of neuritic plaque–negative, tau-positive individuals, a biologically distinct and underrepresented group in Alzheimer’s disease research. The integration of multiple vascular markers and cognitive domains, with adjustment for cardiovascular risk factors, strengthens the validity of the findings and underscores the role of non-amyloid mechanisms in late-life cognitive impairment.

### Future directions

Future research should explore these vascular-cognitive relationships using longitudinal designs to better characterize the temporal dynamics between vascular pathology and cognitive decline in amyloid-negative individuals. In vivo biomarkers of microvascular injury, such as diffusion magnetic resonance imaging or plasma markers of endothelial dysfunction, may help identify at-risk individuals prior to death and facilitate early intervention. Moreover, expanding this work to more racially and ethnically representative populations is essential to understanding how social determinants of health including lifestyle risks may interact with vascular and tau pathology. Finally, incorporating multimodal data including genomics, metabolomics, and inflammation profiles could provide deeper mechanistic insight and identify novel therapeutic targets for individuals on non-AD pathways to dementia.

## Supplementary Information

Below is the link to the electronic supplementary material.

Supplementary Material 1

## Data Availability

All data used in the current analyses is available for download from the NACC (https://www.alz.washington.edu/).

## Abbreviations

A+: amyloid-β (Aβ) plaques
T+: neurofibrillary tau tangles
AD: Alzheimer’s disease
A: amyloid
T: tau
N: neurodegeneration
CAA: cerebral amyloid angiopathy
NACC: National Alzheimer’s Coordinating Center
Aβ: amyloid-β
LBD: Lewy body disease
ADNC: Alzheimer’s disease neuropathologic change
PART: primary age-related tauopathy (PART)
SNAP: suspected non-Alzheimer’s pathophysiology
ADRC: Alzheimer’s Disease Research Centers
MCI: mild cognitive impairment
APOE: apolipoprotein
CAA: cerebral amyloid angiopathy
CADASIL: cerebral autosomal dominant arteriopathy with subcortical infarcts and leukoencephalopathy
FTLD: frontotemporal lobar degeneration
HTN: hypertension
DBT: diabetes
BMI: body mass index
ADSP-PHC: Alzheimer’s Disease Sequencing Project Phenotype Harmonization Consortium
CDR: clinical dementia rating
OR: odds ratio
CI: confidence intervals
CDR-SOB: CDR-Sum of Boxes
β: beta coefficients
