# Supplementary material for: Micro infarcts are associated with cognitive impairment in neurofibrillary tangle predominant decedents: evidence from the NACC autopsy cohort
Source: Alzheimers Res Ther. 2025 Oct 1;17:216. doi: 10.1186/s13195-025-01863-y (PMC12486553; doi:10.1186/s13195-025-01863-y)
Supplement: Supplementary file 1 — Supplementary Material 1 [file 13195_2025_1863_MOESM1_ESM.docx]

# **Supplemental Tables**

| Supplemental Table 1: Primary etiologic diagnoses in amyloid-negative, tau-positive decedents (N = 579) | |
| --- | --- |
| Etiologic Diagnosis | n (%) |
| Alzheimer’s disease (AD) | 226 (39.0) |
| Lewy body disease (LBD) | 78 (13.5) |
| Progressive supranuclear palsy (PSP) | 1 (0.2) |
| Corticobasal degeneration (CBD) | 2 (0.3) |
| Frontotemporal lobar degeneration, other (FTLD-other) | 2 (0.3) |
| Vascular brain injury or vascular dementia (including stroke) | 35 (6.0) |
| Traumatic brain injury (TBI) | 1 (0.2) |
| Normal-pressure hydrocephalus (NPH) | 2 (0.3) |
| Other neurologic, genetic, or infectious condition | 4 (0.7) |
| Depression | 1 (0.2) |
| Schizophrenia or other psychosis | 1 (0.2) |
| Cognitive impairment due to alcohol abuse | 2 (0.3) |
| Cognitive impairment due to other substance abuse | 1 (0.2) |
| Cognitive impairment due to systemic disease or medical illness | 7 (1.2) |
| Cognitive impairment for other specified reasons (e.g., written-in diagnoses) | 10 (1.7) |
| Not cognitively impaired | 185 (32.0) |
| Missing or unknown | 21 (3.6) |
| Diagnoses reflect the most recent adjudicated primary etiologic diagnosis recorded in NACCETPR variable. Participants classified as “Not cognitively impaired” were judged to have normal cognition; "Missing or unknown" indicates no recorded diagnosis. | |

| Supplemental Table 2: Frequency of Neuropathologies Stratified by Braak Stages B2 and B3 | | |
| --- | --- | --- |
| Copathology Burden in Braak B2 Group (n = 452) | | |
| # of Copathologies | Frequency | Percent of Total |
| 0 | 56 | 12.4 % |
| 1 | 115 | 25.4 % |
| 2 | 118 | 26.1 % |
| 3 | 94 | 20.8 % |
| 4 | 48 | 10.6 % |
| 5 | 17 | 3.8 % |
| 6 | 4 | 0.9 % |
| Copathology Burden in Braak B3 Group (n = 62) | | |
| # of Copathologies | Frequency | Percent of Total |
| 0 | 1 | 1.6 % |
| 1 | 11 | 17.7 % |
| 2 | 18 | 29.0 % |
| 3 | 16 | 25.8 % |
| 4 | 9 | 14.5 % |
| 5 | 5 | 8.1 % |
| 6 | 2 | 3.2 % |
| This table summarizes the burden of co-occurring neuropathologies among participants with Braak stage B2 (n = 452) and Braak stage B3 (n = 62). Copathology burden was defined as the number of concurrent pathologies present among the following: neuritic plaques, arteriolosclerosis, atherosclerosis of the circle of Willis, cerebral amyloid angiopathy (CAA), infarcts, and microinfarcts. The table presents the number and percentage of individuals within each Braak group stratified by the count of co-occurring pathologies, ranging from 0 to 6. | | |

| Supplemental Table 3: Pairwise Pearson Correlation Coefficients and Corresponding p-values for Neuropathologies | | | |
| --- | --- | --- | --- |
| Variable 1 | Variable 2 | r | p |
| Neuritic Plaques | Braak (B2) | -0.33 | <0.001 |
| Neuritic Plaques | Braak (B3) | 0.33 | <0.001 |
| Neuritic Plaques | Arteriolosclerosis | -0.02 | 0.673 |
| Neuritic Plaques | Atherosclerosis | -0.09 | 0.028 |
| Neuritic Plaques | CAA | 0.2 | <0.001 |
| Neuritic Plaques | Gross infarcts | 0.04 | 0.307 |
| Neuritic Plaques | Microinfarcts | -0.06 | 0.139 |
| Braak (B2) | Braak (B3) | -1 | <0.001 |
| Braak (B2) | Arteriolosclerosis | -0.03 | 0.446 |
| Braak (B2) | Atherosclerosis | 0 | 0.943 |
| Braak (B2) | CAA | -0.09 | 0.038 |
| Braak (B2) | Gross infarcts | 0 | 0.913 |
| Braak (B2) | Microinfarcts | 0.03 | 0.503 |
| Braak (B3) | Arteriolosclerosis | 0.03 | 0.446 |
| Braak (B3) | Atherosclerosis | 0 | 0.943 |
| Braak (B3) | CAA | 0.09 | 0.038 |
| Braak (B3) | Gross infarcts | 0 | 0.913 |
| Braak (B3) | Microinfarcts | -0.03 | 0.503 |
| Arteriolosclerosis | Atherosclerosis | 0.19 | <0.001 |
| Arteriolosclerosis | CAA | 0.14 | 0.002 |
| Arteriolosclerosis | Gross infarcts | 0.22 | <0.001 |
| Arteriolosclerosis | Microinfarcts | 0.28 | <0.001 |
| Atherosclerosis | CAA | -0.04 | 0.338 |
| Atherosclerosis | Gross infarcts | 0.23 | <0.001 |
| Atherosclerosis | Microinfarcts | 0.15 | <0.001 |
| CAA | Gross infarcts | 0.06 | 0.145 |
| CAA | Microinfarcts | 0.05 | 0.255 |
| Gross infarcts | Microinfarcts | 0.2 | <0.001 |
| Pearson correlation coefficient (r) and two-sided p-value for every pairwise combination of eight binary-coded neuropathologic lesions: neuritic plaques, Braak stage II (B2) and stage III (B3) neurofibrillary tangles, arteriolosclerosis, atherosclerosis, cerebral amyloid angiopathy (CAA), gross infarcts, and microinfarcts. | | | |

| Supplemental Table 4: Cross-validation testing associations between vascular neuropathologies and dementia stages using global clinical dementia rating | | |
| --- | --- | --- |
| Predictor | Base Model OR (95% CI), p | Fully Adjusted OR (95% CI), p |
| Arteriolosclerosis | 1.11 (0.76 – 1.63), 0.58 | 0.95 (0.63–1.42), 0.80 |
| Atherosclerosis | 1.06 (0.73 – 1.55), 0.75 | 0.88 (0.58–1.33), 0.55 |
| CAA | 1.20 (0.76 – 1.87), 0.42 | 1.09 (0.66–1.79), 0.73 |
| Gross Infarcts | **2.02 (1.32 – 3.10), <0.01** | **1.86 (1.16–2.97), 0.01** |
| Microinfarcts | 1.43 (0.96 – 2.12), 0.08 | 1.45 (0.94–2.24), 0.09 |
| Ordinal logistic regression analysis assessing the associations between vascular neuropathologies and dementia severity stages, as measured by the global clinical dementia rating (0/0.5 vs 1 vs 2 vs 3). Odds ratios (OR) with 95% confidence intervals (CI) and corresponding p-values are reported for arteriolosclerosis, atherosclerosis, cerebral amyloid angiopathy (CAA), gross infarcts, and microinfarcts. The model evaluates the likelihood of progressing to higher dementia stages based on the presence of each vascular neuropathology. The base models controlled for age at death (years), years of education, sex, and apolipoprotein ε4 carrier status. The fully adjusted models include all 5 vascular neuropathologies and the covariates. P-values < 0.05 were considered significant. | | |

| Supplementary Table 5: Association between vascular neuropathologies and cognitive domain scores proximate to death controlling for harmonized cardiovascular disease risk | | | |
| --- | --- | --- | --- |
| Predictor | Memory β (95% CI), p | Executive Function β (95% CI), p | Language β (95% CI), p |
| Arteriolosclerosis | –0.12 (–0.34 – 0.10), 0.28 | –0.12 (–0.30 – 0.07), 0.22 | **–0.16 (–0.31 – –0.01), 0.04** |
| Atherosclerosis | –0.08 (–0.31 – 0.16), 0.52 | –0.05 (–0.24 – 0.14), 0.63 | 0.08 (–0.09 – 0.25), 0.36 |
| CAA | –0.15 (–0.44 – 0.14), 0.32 | –0.05 (–0.29 – 0.19), 0.66 | 0.01 (–0.21 – 0.22), 0.96 |
| Gross Infarcts | –0.16 (–0.44 – 0.12), 0.26 | –0.08 (–0.31 – 0.16), 0.52 | –0.16 (–0.36 – 0.05), 0.14 |
| Microinfarcts | **–0.25 (–0.49 – –0.01), 0.04** | **–0.25 (–0.45 – –0.04), 0.02** | **–0.21 (–0.39 – –0.03), 0.02** |
| Linear regression analyses assessing the associations between vascular neuropathologies and cognitive domain scores (memory, executive function, and language) controlling for harmonized cardiovascular disease risk. Beta coefficients (β) with 95% confidence intervals (CI) and corresponding p-values are reported for each vascular neuropathology, including arteriolosclerosis, atherosclerosis, cerebral amyloid angiopathy (CAA), gross infarcts, and microinfarcts. Negative β values indicate worse cognitive performance associated with the presence of the pathology. Statistical significance was defined as p < 0.05. | | | |

| Supplementary Table 6: Association between vascular neuropathologies and cognitive domain scores approximate to death in those without low neuritic plaques | | | |
| --- | --- | --- | --- |
| Predictor | Memory β (95% CI), p | Executive Function β (95% CI), p | Language β (95% CI), p |
| Arteriolosclerosis | –0.12 (–0.42 – 0.17), 0.41 | –0.19 (–0.48 – 0.09), 0.19 | –0.05 (–0.29 – 0.19), 0.67 |
| Atherosclerosis | –0.19 (–0.49 – 0.11), 0.22 | –0.17 (–0.46 – 0.12), 0.24 | 0.12 (–0.12 – 0.36), 0.32 |
| CAA | –0.22 (–0.74 – 0.30), 0.40 | –0.16 (–0.64 – 0.32), 0.51 | 0.27 (–0.16 – 0.71), 0.22 |
| Gross Infarcts | **–0.38 (–0.75 – –0.01), 0.04** | –0.17 (–0.57 – 0.23), 0.41 | –0.28 (–0.58 – 0.02), 0.07 |
| Microinfarcts | **–0.41 (–0.72 – –0.11), 0.01** | **–0.48 (–0.77 – –0.19), <0.01** | **–0.27 (–0.51 – –0.02), 0.03** |
| Linear regression analyses examining the associations between vascular neuropathologies and cognitive domain scores (memory, executive function, and language), after excluding individuals with low neuritic amyloid pathology (C1). Beta coefficients (β) with 95% confidence intervals (CI) and corresponding p-values are reported for arteriolosclerosis, atherosclerosis, cerebral amyloid angiopathy (CAA), gross infarcts, and microinfarcts. Negative β values indicate poorer cognitive performance associated with the presence of the neuropathology. Statistical significance was defined as p < 0.05. | | | |

| Supplementary Table 7: Association between vascular neuropathologies and cognitive domain scores adjusting for neuritic plaques and Braak stage | | | |
| --- | --- | --- | --- |
| Predictor | Memory β (95% CI), p | Executive Function β (95% CI), p | Language β (95% CI), p |
| Arteriolosclerosis | –0.09 (–0.30 – 0.11), 0.37 | –0.11 (–0.28 – 0.08), 0.25 | –0.12 (–0.27 – 0.02), 0.10 |
| Atherosclerosis | –0.11 (–0.32 – 0.10), 0.31 | –0.04 (–0.23 – 0.14), 0.64 | 0.06 (–0.10 – 0.22), 0.45 |
| CAA | –0.05 (–0.32 – 0.22), 0.71 | –0.01 (–0.25 – 0.23), 0.95 | 0.12 (–0.08 – 0.32), 0.25 |
| Gross Infarcts | –0.24 (–0.48 – 0.01), 0.06 | –0.05 (–0.28 – 0.18), 0.65 | –0.16 (–0.35 – 0.03), 0.10 |
| Microinfarcts | **–0.26 (–0.48 – –0.05), 0.02** | **–0.25 (–0.44 – –0.05), 0.01** | **–0.22 (–0.38 – –0.05), 0.01** |
| Linear regression analyses examining the associations between vascular neuropathologies and cognitive domain scores (memory, executive function, and language) controlling for neuritic plaques (C0 vs C1) and Braak stage (B2 vs. B3). Beta coefficients (β) with 95% confidence intervals (CI) and corresponding p-values are reported for arteriolosclerosis, atherosclerosis, cerebral amyloid angiopathy (CAA), gross infarcts, and microinfarcts. Negative β values indicate poorer cognitive performance associated with the presence of the neuropathology. Statistical significance was defined as p < 0.05. | | | |

| Supplementary Table 8: Association between vascular neuropathologies and cross-sectional cognitive domain scores adjusting for neuritic plaques and individual Braak levels | | | | | | |
| --- | --- | --- | --- | --- | --- | --- |
| Cognitive Domain | Predictor | Arteriolosclerosis β (95% CI), p | Atherosclerosis β (95% CI), p | CAA β (95% CI), p | Gross Infarcts β (95% CI), p | Microinfarcts β (95% CI), p |
| Memory | Neuritic Plaque (C1 vs C0) | −0.14 (−0.36 – 0.08), 0.21 | −0.18 (−0.40 – 0.04), 0.11 | −0.13 (−0.35 – 0.09), 0.24 | −0.14 (−0.36 – 0.08), 0.22 | −0.16 (−0.38 – 0.05), 0.14 |
|  | Braak IV vs Braak III | −0.10 (−0.32 – 0.13), 0.39 | −0.17 (−0.39 – 0.06), 0.14 | −0.19 (−0.41 – 0.03), 0.10 | −0.19 (−0.41 – 0.04), 0.10 | −0.19 (−0.41 – 0.03), 0.09 |
|  | Braak V vs Braak III | **−0.62 (−1.06 – −0.17), 0.01** | **−0.95 (−1.35 – −0.55), 0.01** | **−1.03 (−1.42 – −0.64), 0.01** | **−1.01 (−1.40 – −0.62), 0.01** | **−0.98 (−1.36 – −0.59), 0.01** |
|  | Braak VI vs Braak III | **−0.70 (−1.23 – −0.17), 0.01** | **−1.12 (−1.55 – −0.69), 0.01** | **−1.16 (−1.58 – −0.74), 0.01** | **−1.18 (−1.60 – −0.76), 0.01** | **−1.19 (−1.60 – −0.77), 0.01** |
|  | Vascular Predictor | −0.09 (−0.30 – 0.11), 0.38 | −0.09 (−0.30 – 0.12), 0.40 | −0.05 (−0.32 – 0.22), 0.71 | −0.22 (−0.47 – 0.03), 0.08 | **−0.27 (−0.48 – −0.06), 0.01** |
| Executive Function | Neuritic Plaque (C1 vs C0) | −0.12 (−0.31 – 0.08), 0.24 | −0.16 (−0.36 – 0.03), 0.10 | −0.15 (−0.35 – 0.04), 0.12 | −0.15 (−0.34 – 0.04), 0.13 | −0.17 (−0.36 – 0.02), 0.08 |
|  | Braak IV vs Braak III | 0.04 (−0.16 – 0.24), 0.68 | −0.01 (−0.21 – 0.19), 0.93 | −0.01 (−0.21 – 0.18), 0.91 | −0.01 (−0.20 – 0.19), 0.95 | −0.01 (−0.21 – 0.18), 0.91 |
|  | Braak V vs Braak III | 0.11 (−0.29 – 0.52), 0.58 | 0.01 (−0.39 – 0.40), 0.98 | −0.04 (−0.42 – 0.34), 0.84 | −0.05 (−0.42 – 0.33), 0.80 | −0.03 (−0.40 – 0.34), 0.86 |
|  | Braak VI vs Braak III | −0.13 (−0.64 – 0.38), 0.62 | −0.42 (−0.84 – −0.01), 0.05 | **−0.43 (−0.85 – −0.02), 0.04** | −0.40 (−0.80 – 0.01), 0.06 | −0.40 (−0.80 – −0.00), 0.05 |
|  | Vascular Predictor | −0.10 (−0.29 – 0.08), 0.26 | −0.04 (−0.23 – 0.14), 0.64 | −0.01 (−0.25 – 0.23), 0.96 | −0.03 (−0.27 – 0.20), 0.78 | **−0.25 (−0.44 – −0.05), 0.01** |
| Language | Neuritic Plaque (C1 vs C0) | **−0.16 (−0.32 – −0.01), 0.04** | **−0.17 (−0.34 – −0.01), 0.04** | **−0.19 (−0.35 – −0.02), 0.03** | **−0.18 (−0.34 – −0.02), 0.03** | **−0.19 (−0.35 – −0.03), 0.02** |
|  | Braak IV vs Braak III | −0.01 (−0.17 – 0.14), 0.86 | −0.09 (−0.25 – 0.08), 0.30 | −0.09 (−0.26 – 0.07), 0.26 | −0.08 (−0.25 – 0.08), 0.33 | −0.09 (−0.25 – 0.08), 0.29 |
|  | Braak V vs Braak III | 0.22 (−0.12 – 0.56), 0.20 | −0.22 (−0.55 – 0.10), 0.18 | −0.29 (−0.61 – 0.03), 0.08 | −0.26 (−0.58 – 0.06), 0.11 | −0.25 (−0.56 – 0.07), 0.12 |
|  | Braak VI vs Braak III | **−0.50 (−0.87 – −0.13), 0.01** | **−0.80 (−1.11 – −0.48), 0.01** | **−0.82 (−1.13 – −0.51), 0.01** | **−0.83 (−1.14 – −0.51), 0.01** | **−0.84 (−1.15 – −0.54), 0.01** |
|  | Vascular Predictor | −0.12 (−0.26 – 0.02), 0.10 | 0.08 (−0.08 – 0.24), 0.31 | 0.12 (−0.08 – 0.32), 0.23 | −0.13 (−0.32 – 0.06), 0.17 | **−0.22 (−0.38 – −0.06), 0.01** |
| This table presents the results of linear regression analyses examining the associations between vascular neuropathologies and cognitive domain scores (memory, executive function, and language) for neuritic plaques (C0 vs C1) and individual Braak staging (III, IV, V, VI). Beta coefficients (β) with 95% confidence intervals (CI) and corresponding p-values are reported for arteriolosclerosis, atherosclerosis, cerebral amyloid angiopathy (CAA), gross infarcts, and microinfarcts. Negative β values indicate poorer cognitive performance associated with the presence of the pathology. Statistical significance was defined as 0.05. | | | | | | |

| Supplemental Table 9: Association between vascular neuropathologies and cross-sectional cognitive domain scores proximal to death controlling for the time between the last visit and death | | | |
| --- | --- | --- | --- |
| Predictor | Memory β (95% CI), p | Executive Function β (95% CI), p | Language β (95% CI), p |
| Arteriolosclerosis | –0.12 (–0.33 – 0.09), 0.25 | –0.11 (–0.30 – 0.07), 0.22 | –0.14 (–0.28 – 0.01), 0.07 |
| Atherosclerosis | –0.11 (–0.33 – 0.11), 0.33 | –0.03 (–0.22 – 0.15), 0.74 | 0.07 (–0.09 – 0.24), 0.36 |
| CAA | –0.20 (–0.48 – 0.08), 0.15 | –0.07 (–0.30 – 0.17), 0.59 | 0.01 (–0.19 – 0.22), 0.89 |
| Gross Infarcts | –0.21 (–0.48 – 0.05), 0.11 | –0.07 (–0.30 – 0.16), 0.54 | –0.17 (–0.37 – –0.02), 0.08 |
| Microinfarcts | **–0.29 (–0.52 – –0.07), 0.01** | **–0.24 (–0.44 – –0.04), 0.02** | **–0.21 (–0.38 – –0.04), 0.01** |
| This table presents the results of linear regression analyses examining the associations between vascular neuropathologies and cognitive domain scores (memory, executive function, and language) in the period proximal to death when controlling for the time between the last visit and death. The table reports beta coefficients (β) with 95% confidence intervals (CI) and corresponding p-values for each vascular neuropathology. Negative β values indicate worse cognitive performance associated with the presence of the pathology. Statistical significance was defined as p < 0.05. | | | |

| Supplemental Table 10: Association between all vascular neuropathologies and cross-sectional cognitive domain scores proximal to death | | | |
| --- | --- | --- | --- |
| Predictor | Memory β (95% CI), p | Executive Function β (95% CI), p | Language β (95% CI), p |
| Arteriolosclerosis | -0.04 (-0.26 – 0.18), 0.73 | –0.06 (–0.25 – 0.13), 0.56 | –0.11 (–0.26 – 0.05), 0.17 |
| Atherosclerosis | -0.05 (-0.27 – 0.17), 0.67 | –0.02 (–0.21 – 0.17), 0.81 | 0.13 (–0.02 – 0.29), 0.09 |
| CAA | -0.00 (-0.29 – 0.28), 0.99 | 0.09 (–0.16 – 0.33), 0.49 | 0.15 (–0.05 – 0.35), 0.14 |
| Gross Infarcts | -0.18 (-0.44 – 0.08), 0.16 | –0.05 (–0.28 – 0.18), 0.67 | –0.17 (–0.35 – 0.01), 0.07 |
| Microinfarcts | **-0.30 (-0.54 – -0.07), 0.01** | **–0.26 (–0.46 – –0.05), 0.02** | **–0.17 (–0.33 – –0.00), 0.04** |
| Linear regression analyses examining the associations between a model including all five vascular neuropathologies and cognitive domain scores (memory, executive function, and language) in the period proximal to death. The table reports beta coefficients (β) with 95% confidence intervals (CI) and corresponding p-values for each vascular neuropathology. Negative β values indicate worse cognitive performance associated with the presence of the pathology. Statistical significance was defined as p < 0.05. | | | |

| Supplemental Table 11: Association between all vascular neuropathologies and cross-sectional cognitive domain scores proximal to death when controlling time between the last visit and death | | | |
| --- | --- | --- | --- |
| Predictor | Memory β (95% CI), p | Executive Function β (95% CI), p | Language β (95% CI), p |
| Arteriolosclerosis | -0.04 (-0.26 – 0.18), 0.72 | -0.06 (-0.25 – 0.13), 0.55 | -0.11 (-0.26 – 0.05), 0.17 |
| Atherosclerosis | -0.05 (-0.26 – 0.17), 0.68 | -0.02 (-0.21 – 0.17), 0.82 | 0.13 (-0.02 – 0.29), 0.09 |
| CAA | -0.04 (-0.32 – 0.24), 0.77 | 0.08 (-0.17 – 0.32), 0.52 | 0.14 (-0.06 – 0.34), 0.16 |
| Gross Infarcts | -0.16 (-0.42 – 0.09), 0.21 | -0.04 (-0.28 – 0.19), 0.71 | -0.17 (-0.35 – 0.02), 0.08 |
| Microinfarcts | **-0.32 (-0.55 – -0.08), 0.01** | **-0.26 (-0.47 – -0.05), 0.01** | **-0.17 (-0.34 – -0.01), 0.04** |
| Linear regression analyses examining the associations between a model including all five vascular neuropathologies and cognitive domain scores (memory, executive function, and language) in the period proximal to death when controlling for the time between the last visit and death. The table reports beta coefficients (β) with 95% confidence intervals (CI) and corresponding p-values for each vascular neuropathology. Negative β values indicate worse cognitive performance associated with the presence of the pathology. Statistical significance was defined as p < 0.05. | | | |

| Supplementary Table 12: Interaction effects between vascular neuropathologies and years to death on longitudinal cognitive outcomes in those without neuritic plaques | | | |
| --- | --- | --- | --- |
| Predictors | Memory β (95% CI), p | Executive Function β (95% CI), p | Language β (95% CI), p |
| Years to death × Arteriolosclerosis | 0.01 (–0.02 – 0.03), 0.63 | -0.01 (–0.04 – 0.01), 0.99 | 0.00 (–0.02 – 0.02), 0.69 |
| Years to death × Atherosclerosis | -0.01 (–0.04 – 0.02), 0.42 | 0.01 (-0.01 – 0.04), 0.21 | 0.00 (–0.02 – 0.02), 0.68 |
| Years to death × CAA | 0.02 (–0.02 – 0.07), 0.32 | –0.02 (–0.06 – 0.02), 0.23 | 0.03 (–0.01 – 0.06), 0.12 |
| Years to death × Gross Infarcts | –0.03 (–0.06 – 0.01), 0.09 | 0.01 (–0.02 – 0.04), 0.60 | –0.01 (–0.04 – 0.01), 0.37 |
| Years to death × Microinfarcts | -0.01 (–0.04 – 0.01), 0.29 | -0.02 (–0.04 – 0.01), 0.16 | –0.01 (–0.03 – 0.01), 0.18 |
| Linear mixed-effects models were used to evaluate the interaction between time to death and each vascular neuropathologies (arteriolosclerosis, atherosclerosis, cerebral amyloid angiopathy (CAA), gross infarcts, and microinfarcts) on cognitive trajectories in those without neuritic plaques. Outcomes included memory, executive function, and language. Interaction terms (years to death × vascular neuropathology) reflect differences in the rate of cognitive decline associated with each neuropathological marker. Estimates reflect the slope difference in cognitive change over time associated with each vascular neuropathology. Beta coefficients (β), 95% confidence intervals (CI), and p-values are reported for each domain. Significant associations (p < 0.05) are bolded. | | | |

**
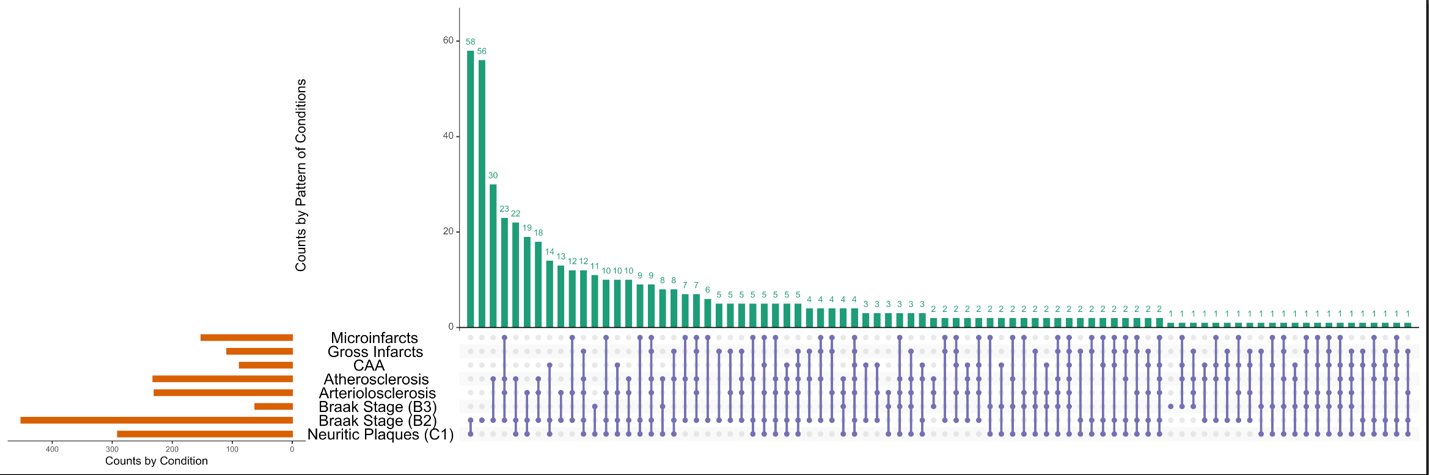
**

**Supplemental Figure 1. Prevalence and Co-occurrence of Neuropathologies in the Selected Sample**

**Supplemental Figure 1 Legend.** The **left horizontal bar chart** shows the **marginal frequency** of each neuropathology in the cross-sectional sample (orange bars): neuritic plaques, Braak neurofibrillary tangle stages II (B2) and III (B3), arteriolosclerosis, atherosclerosis, cerebral amyloid angiopathy (CAA), gross infarcts, and microinfarcts. The **right panel** is an UpSet plot of the **intersection frequencies** (teal bars): each bar’s height is the number of participants exhibiting exactly that combination of pathologies. In the matrix below, **filled purple circles** denote presence of that neuropathology in the combination, **light‐gray circles** denote absence, and **connecting lines** group the circles into the same intersection set. The UpSet plot shows all combinations of neuropathologies.
